# Supplementary material for: Neighborhood and Child Development at Age Five: A UK–US Comparison
Source: Int J Environ Res Public Health. 2021 Oct 4;18(19):10435. doi: 10.3390/ijerph181910435 (PMC8508230; doi:10.3390/ijerph181910435)
Supplement: Supplementary file 1 [file ijerph-18-10435-s001.zip › ijerph-1383923-supplementary.pdf]

Supplemental Material

Contents

Supplement S1:

- Additional Descriptive Statistics S--2

Supplement S2:

- Full Tables for Multivariate Analyses S--7

Supplement S3:

- Plots of Predicted Margins on "Social Ecology and Mobility" Variables S-16

Supplement S4:

- Sensitivity Analyses S-17

Supplement S1: Additional Descriptive Statistics

Table S1.1 Weighted Univariate Statistics of Variables for Sensitivity Analyses in UK and US

Variable	UK				US			
	N	***Mean***	***SD***	***Range***	***N***	***Mean***	***SD***	***Range***
Area social advantage at 1yr (original score)	7,967	-0.1	1.1	-3.9-2.4	1,820	-0.5	1.3	-5.7-2.0
Area social advantage at 1yr (original score) for movers only	*3,138*	*-0.1*	*1.1*	*-3.9-2.2*	*1,228*	*-0.5*	*1.2*	*-5.7-2.0*
Change in area social advantage 1-5yrs (difference of original scores)	7,967	0.0	0.6	-3.9-4.5	1,820	0.4	0.9	-3.8-4.5
Change in area social advantage 1-5yrs (difference of original scores) for movers only	*3,138*	*0.1*	*0.9*	*-3.9-4.5*	*1,228*	*0.1*	*1.1*	*-3.8-4.5*
LN area social advantage at 1yr (natural log)	7,967	1.3	0.4	-4.6-1.8	1,820	1.6	0.3	-4.6-2.0
Change in LN area social advantage 1-5yrs (difference of LNs)	7,967	0.0	0.2	-5.3-5.3	1,820	-0.1	0.2	-1.9-5.3
Negative change in area soc. advantage 1-5yrs (diff. of percentiles, positive values = 0)	7,967	-3.1	9.6	-91-0	1,820	-5.5	12.5	-76-0
Negative change in area soc. advantage 1-5yrs (diff. of percentiles, positive values = 0) for movers only	*3,138*	*-7.9*	*13.9*	*-91-0*	*1,228*	*-8.7*	*14.8*	-76-0
Positive change in area social advantage 1-5yrs (difference of percentiles, negative values = 0)	7,967	3.8	11.0	0-95	1,820	5.5	12.9	0-92
Positive change in area social advantage 1-5yrs (difference of percentiles, negative values = 0) for movers only	*3,138*	*9.5*	*15.8*	*0-95*	*1,228*	*8.8*	*15.4*	0-92

Table S1.2 Pearson's Correlations[a] in UK Sample (extended)[b]

Variables	(1)	(2)	(3)	(4)	(5)	(6)	(7)	(8)	(9)	(10)	(11)	(12)	(13)	(14)	(15)	(16)	(17)	(18)	(19)	(20)	(21)	(22)	(23)	(24)	(25)	(26)	(27)	(28)	(29)	(30)	(31)	(32)	(33)	(34)	(35)	(36)	(37)	(38)	(39)	(40)	(41)	(42)	(43)
(1) Verbal score at 5yrs (percentile)	—																																										
(2) Externalizing behavior adjustment at 5 yrs (percentile)	.20	—																																									
(3) Internalizing behavior adjustment at 5yrs (percentile)	.16	.34	—																																								
(4) Area social advantage at 1yr (percentile)	.32	.21	.21	—																																							
(5) Area social advantage at 5yrs (percentile)	.31	.20	.20	.83	—																																						
(6) Change in area social advantage 1-5yrs (difference of percentiles)	.00 NS	-.02	-.01 NS	-.27	.26	—																																					
(7) Moved between 1-5yrs	-.01 NS	-.04	-.03	-.02	.02	.05	—																																				
(8) Household was workless at 1yr	-.23	-.18	-.16	-.35	-.36	-.02 NS	.08	—																																			
(9) Mother was single at 1yr	-.13	-.15	-.12	-.27	-.28	-.03	.10	.66	—																																		
(10) LN equivalized income at 1yr	.35	.23	.22	.56	.56	.00 NS	-.07	-.59	-.46	—																																	
(11) Mother's level of education	.36	.26	.22	.48	.48	.01 NS	.00 NS	-.37	-.26	.54	—																																
(12) White	.29	.03	.12	.15	.15	.00 NS	.04	-.09	-.04	.21	.16	—																															
(13) Black	-.13	.00 NS	-.03	-.07	-.07	.01 NS	-.01 NS	.09	.14	-.09	-.03	-.47	—																														
(14) Indian	-.05	-.01 NS	-.02	-.01 NS	.01 NS	.02 NS	-.03	-.02	-.04	-.01 NS	-.02 NS	-.41	-.03	—																													
(15) Pakistani/ Bangladeshi	-.24	-.05	-.12	-.17	-.17	.00 NS	-.03	.05	-.05	-.20	-.19	-.56	-.04	-.04	—																												
(16) Other race/ethnicity	-.10	.00 NS	-.05	-.01 NS	-.02 NS	-.03	.00 NS	.04	.02 NS	-.06	-.03	-.43	-.03	-.03	-.04	—																											
(17) Mother was not born in UK	-.20	.00 NS	-.06	-.03	-.04	-.02 NS	-.03	.06	-.02 NS	-.11	-.13	-.56	.20	.18	.37	.28	—																										
(18) Household size at 1yr	-.22	-.01 NS	-.04	-.11	-.12	-.02	-.07	.00 NS	-.15	-.19	-.20	-.23	.01	.10	.26	.02	.16	—																									
(19) Mother's age in years at cohort member's birth	.18	.19	.14	.38	.38	.00 NS	-.19	-.32	-.29	.41	.30	.03	.07	-.01	-.10	.01 NS	.03	.14	—																								
(20) Cohort member was first child	.15	.00 NS	-.05	.07	.07	.00 NS	.15	-.02 NS	.05	.12	.12	.05	-.04	-.01 NS	-.05	.01 NS	-.04	-.53	-.27	—																							
(21) Public housing	-.24	-.20	-.17	-.45	-.42	.04	.03	.47	.36	-.50	-.38	-.10	.19	-.05	.01 NS	.04	.05	.02 NS	-.28	-.11	—																						
(22) Subsidized rented housing	-.07	-.05	-.01 NS	-.07	-.10	-.06	.08	.22	.14	-.18	-.08	.00	-.01	-.01 NS	.00 NS	.02 NS	.02 NS	-.05	-.10	.01 NS	-.09	—																					
(23) Market rented housing	.00 NS	-.02 NS	.00 NS	.03	-.01 NS	-.06	.13	-.02 NS	-.01 NS	-.02 NS	-.01 NS	-.01 NS	-.02 NS	.00 NS	.00 NS	.05	.03	-.06	-.07	.06	-.12	-.03	—																				
(24) Owned housing	.27	.22	.17	.44	.45	.03	-.17	-.53	-.45	.61	.42	.15	-.14	.01 NS	-.08	-.07	-.09	-.10	.43	.01 NS	-.73	-.22	-.30	—																			
(25) Shared/other types of housing	-.08	-.05	-.04	-.08	-.10	-.04	.12	.12	.19	-.21	-.12	-.12	-.02 NS	.08	.15	.01 NS	.06	.26	-.25	.13	-.13	-.04	-.05	-.33	—																		
(26) Cohort member was born underweight	-.05	-.06	-.06	-.03	-.04	-.01 NS	.02 NS	.04	.01 NS	-.04	-.07	-.07	.02	.05	.05	.01 NS	.03	.00 NS	-.02	.05	.03	.02 NS	.03	-.04	.00 NS	—																	
(27) Cohort member's gen. health at 3yrs	-.04	-.05	-.04	-.04	-.04	.01 NS	.01 NS	.01 NS	.03	-.02 NS	.00 NS	.04	-.02 NS	-.01 NS	-.04	.00 NS	-.05	-.03	-.03	.00 NS	.03	.00 NS	.02 NS	-.04	.00 NS	.05	—																

Variables	(1)	(2)	(3)	(4)	(5)	(6)	(7)	(8)	(9)	(10)	(11)	(12)	(13)	(14)	(15)	(16)	(17)	(18)	(19)	(20)	(21)	(22)	(23)	(24)	(25)	(26)	(27)	(28)	(29)	(30)	(31)	(32)	(33)	(34)	(35)	(36)	(37)	(38)	(39)	(40)	(41)	(42)	(43)
**(28) Mother's general health at 1yr**	.12	.17	.17	.19	.21	.02 NS	-.02 NS	-.15	-.09	.23	.18	.10	-.02	-.04	-.07	-.04	-.06	-.09	.09	.08	-.18	-.04	.02 NS	.18	-.03	-.07	-.05	—															
**(29) Mother depressed at 1yr**	-.04	-.11	-.10	-.10	-.11	-.01 NS	.04	.11	.08	-.13	-.11	.09	-.05	-.05	-.03	-.03	-.07	.03	-.04	-.08	.11	.05	.02 NS	-.11	-.03	.04	.06	-.25	—														
**(30) Household was workless at 5yrs**	-.08	-.07	-.05	-.11	-.10	.02 NS	.03	-.10	.01 NS	-.12	-.13	-.04	.03	-.02 NS	.05	.02 NS	.02 NS	.07	-.12	-.01 NS	.14	.04	.03	-.17	.04	.01 NS	.02 NS	-.07	.07	—													
**(31) Household was employed at 5yrs**	-.09	-.09	-.07	-.17	-.17	-.01 NS	.05	.60	.40	-.35	-.16	-.04	.01 NS	.00 NS	.05	.02 NS	.04	-.03	-.20	.01 NS	.24	.15	.00 NS	-.30	.09	.02 NS	.00 NS	-.05	.06	-.06	—												
**(32) Mother was single at 5yrs**	-.06	-.09	-.06	-.09	-.09	.00 NS	.06	-.01 NS	-.12	-.09	-.08	.00 NS	.03	-.03	-.01 NS	.01 NS	-.04	.03	-.10	.00 NS	.11	.03	.03	-.12	-.01 NS	.02 NS	.01 NS	-.07	.08	.37	-.06	—											
**(33) Mother was coupled at 5yrs**	-.06	-.09	-.05	-.15	-.16	-.02 NS	.07	.35	.56	-.25	-.14	.01 NS	.03	-.02	-.02 NS	-.01 NS	-.02 NS	-.09	-.20	.04	.18	.07	.02 NS	-.25	.11	-.01 NS	.01 NS	-.04	.04	-.03	.44	-.07	—										
**(34) Change in LN equivalized income 1-5yrs**	-.04	-.02 NS	.00 NS	-.09	-.08	.02 NS	.02	.17	.14	-.44	-.06	-.01 NS	-.01 NS	.02 NS	.00 NS	.02 NS	-.01 NS	.02	-.08	-.05	.08	.04	-.01 NS	-.12	.09	-.01 NS	.00 NS	-.02	.02 NS	-.18	.23	-.19	.17	—									
**(35) Change in household size 1-5yrs**	.06	.04	.03	.08	.10	.04	.00 NS	-.02 NS	.00 NS	.12	.10	.05	.00 NS	-.02 NS	-.04	-.02 NS	.02 NS	-.45	-.01 NS	.16	.02 NS	.04	.03	.10	-.29	.00	-.02 NS	.08	-.05	-.13	.04	-.31	.14	-.02	—								
**(36) Cohort member's sex in male**	-.04	-.12	.00 NS	.00 NS	-.01 NS	-.01 NS	-.01 NS	.00 NS	.01 NS	.00 NS	-.01 NS	.00 NS	-.01 NS	.02 NS	-.01 NS	-.01 NS	.01 NS	.02 NS	.00 NS	-.01 NS	-.02 NS	.00 NS	.03	-.01 NS	.02	-.02 NS	.02 NS	.01 NS	.00 NS	-.02 NS	.00 NS	.00 NS	.01 NS	-.01 NS	-.01 NS	—							
**(37) Cohort member's age in months at 5yrs**	-.02	.03	.02 NS	.04	.05	.02 NS	.05	.02 NS	-.01 NS	.00 NS	.02 NS	.00 NS	.00 NS	.01 NS	-.01 NS	-.01 NS	.01 NS	.00 NS	-.01 NS	.01 NS	-.02 NS	.03	.00 NS	.00 NS	.00 NS	-.04	-.01 NS	.03	-.02 NS	.00 NS	.03	.04	-.01 NS	.00 NS	.01 NS	.02 NS	—						
(38) Area social advantage at 1yr (original score)	*.31*	*.21*	*.20*	*.98*	*.81*	*-.27*	*-.02 NS*	*-.36*	*-.27*	*.56*	*.48*	*.15*	*-.06*	*.00 NS*	*-.18*	*.00 NS*	*-.03*	*-.11*	*.38*	*.07*	*-.46*	*-.08*	*.03*	*.45*	*-.08*	*-.03*	*-.04*	*.19*	*-.10*	*-.11*	*-.18*	*-.09*	*-.15*	*-.09*	*.08*	*.00 NS*	*.04*	—					
(39) Change area social advantage 1-5yrs difference of original scores)	*-.01*	*-.03*	*-.01 NS*	*-.28*	*.24*	*.97*	*.10*	*-.01 NS*	*-.02 NS*	*-.01 NS*	*.00 NS*	*-.01 NS*	*.01 NS*	*.02 NS*	*.01 NS*	*-.03*	*-.01 NS*	*-.02*	*-.02*	*.01 NS*	*.06*	*-.06*	*-.06*	*.01 NS*	*-.03*	*-.01 NS*	*.02 NS*	*.01 NS*	*.00 NS*	*.03*	*.00 NS*	*.01 NS*	*-.01 NS*	*.02 NS*	*.03*	*-.01 NS*	*.02 NS*	*-.29*	—				
(40) LN area social advantage (natural log)	*.30*	*.20*	*.19*	*.92*	*.76*	*-.25*	*-.03*	*-.37*	*-.27*	*.52*	*.45*	*.13*	*-.04*	*.01 NS*	*-.18*	*.00 NS*	*-.04*	*-.11*	*.36*	*.06*	*-.45*	*-.07*	*.03*	*.44*	*-.08*	*-.03*	*-.04*	*.18*	*-.10*	*-.11*	*-.18*	*-.08*	*-.15*	*-.08*	*.06*	*.00 NS*	*.03*	*.96*	*-.29*	—			
(41) Change LN area social advan. 1-5yrs (difference of LNs)	*-.02 NS*	*-.02 NS*	*-.01 NS*	*-.25*	*.19*	*.82*	*.09*	*.00 NS*	*-.01 NS*	*-.02 NS*	*-.01 NS*	*-.01 NS*	*.01 NS*	*.01 NS*	*.01 NS*	*-.02 NS*	*-.01 NS*	*-.02 NS*	*-.02*	*.01 NS*	*.07*	*-.06*	*-.05*	*-.01 NS*	*-.03*	*.00 NS*	*.02 NS*	*.00 NS*	*.00 NS*	*.03*	*.01 NS*	*.01 NS*	*.00 NS*	*.02 NS*	*.02*	*-.01 NS*	*.02 NS*	*-.28*	*.92*	*-.31*	—		
(42) Negative change area social advantage 1-5yrs (diff. p-tiles, p.=0)	*-.01 NS*	*.00 NS*	*.00 NS*	*-.19*	*.18*	*.70*	*-.40*	*-.04*	*-.06*	*.04*	*.00 NS*	*-.01 NS*	*.01 NS*	*.04*	*.02*	*-.04*	*-.02 NS*	*.02 NS*	*.08*	*-.08*	*.02 NS*	*-.10*	*-.13*	*.11*	*-.09*	*-.02 NS*	*.00 NS*	*.02*	*-.02*	*-.02 NS*	*-.03*	*-.03*	*-.04*	*.00 NS*	*.04*	*.00 NS*	*-.01 NS*	*-.19*	*.66*	*-.18*	*.55*	—	
(43) Positive change area social advantage 1-5yrs (diff.. p-tile, n.=0)	*.00*	*-.03*	*-.01*	*-.21*	*.22*	*.79*	*.42*	*.01 NS*	*.02 NS*	*-.03*	*.02 NS*	*.01 NS*	*.01 NS*	*.00 NS*	*-.02 NS*	*-.01 NS*	*-.01 NS*	*-.05*	*-.08*	*.07*	*.04*	*.00 NS*	*.02 NS*	*-.05*	*.02 NS*	*.00 NS*	*.01 NS*	*.01 NS*	*.01 NS*	*.04*	*.01 NS*	*.03*	*.02 NS*	*.02 NS*	*.02 NS*	*-.02 NS*	*.03*	*-.21*	*.78*	*-.19*	*.67*	*.11*	—

[a] Correlations are statistically significant at least at $p < .05$ unless noted as "Not (statistically) Significant" (NS).

[b] Additional variables used in the sensitivity analyses are listed in *italics* at the bottom of the table (see row 38ff).

Table S1.3 Pearson's Correlations[a] in US Sample (extended)[b]

Variables	(1)	(2)	(3)	(4)	(5)	(6)	(7)	(8)	(9)	(10)	(11)	(12)	(13)	(14)	(15)	(16)	(17)	(18)	(19)	(20)	(21)	(22)	(23)	(24)	(25)	(26)	(27)	(28)	(29)	(30)	(31)	(32)	(33)	(34)	(35)	(36)	(37)	(38)	(39)	(40)	(41)	(42)
**(1) Verbal score at 5yrs (percentile)**	—																																									
**(2) Externalizing behavior adjustment at 5 yrs (percentile)**	.17	—																																								
**(3) Internalizing behavior adjustment at 5yrs (percentile)**	.27	.57	—																																							
**(4) Area social advantage at 1yr (percentile)**	.44	.16	.16	—																																						
**(5) Area social advantage at 5yrs (percentile)**	.52	.16	.19	.78	—																																					
**(6) Change in area social advantage 1-5yrs (difference of percentiles)**	.12	.00 NS	.05	-.31	.36	—																																				
**(7) Moved between 1-5yrs**	.00 NS	.06	-.02 NS	-.02 NS	.00 NS	.00 NS	—																																			
**(8) Household was workless at 1yr**	-.20	-.09	-.07	-.34	-.27	.10	.04 NS	—																																		
**(9) Mother was single at 1yr**	-.23	-.10	-.06	-.30	-.24	.08	.06	.50	—																																	
**(10) LN equivalized income at 1yr**	.47	.14	.18	.54	.53	.00 NS	-.05 NS	-.39	-.34	—																																
**(11) Mother's level of education**	.50	.14	.18	.52	.56	.07	-.05 NS	-.27	-.25	.61	—																															
**(12) White**	.53	.11	.09	.52	.53	.03 NS	.00 NS	-.18	-.18	.40	.37	—																														
**(13) Black**	-.24	-.11	-.01 NS	-.41	-.39	.03 NS	-.03 NS	.24	.30	-.24	-.11	-.45	—																													
**(14) Hispanic**	-.36	.03 NS	-.10	-.26	-.28	-.03 NS	.01 NS	.02 NS	-.03 NS	-.28	-.36	-.52	-.37	—																												
**(15) Other race/ethnicity**	.07	-.07	.02 NS	.19	.14	-.06	.04 NS	-.10	-.11	.15	.13	-.22	-.16	-.18	—																											
**(16) Mother was not born in US**	-.26	-.03 NS	-.10	-.14	-.10	.06	-.06	-.04 NS	-.04 NS	-.16	-.24	-.39	-.19	.44	.27	—																										
**(17) Household size at 1yr**	-.28	-.11	-.07	-.26	-.26	.00 NS	-.07	.05	.10	-.30	-.33	-.22	.11	.15	-.04 NS	.10	—																									
**(18) Mother's age in years at cohort member's birth**	.28	.16	.07	.29	.34	.09	-.17	-.28	-.31	.30	.45	.16	-.17	-.05	.07	.12	-.04 NS	—																								
**(19) Cohort member was first child**	.08	.04 NS	.02 NS	.14	.11	-.05	.14	.04 NS	.12	.15	.08	.13	-.08	-.07	.00 NS	-.09	-.38	-.32	—																							
**(20) Public housing**	-.19	-.09	-.05	-.31	-.28	.03 NS	-.04 NS	.21	.12	-.22	-.14	-.21	.33	-.05	-.06	-.10	.05	-.15	-.13	—																						
**(21) Subsidized rented housing**	-.16	-.09	-.07	-.16	-.10	.08	-.03 NS	.18	.10	-.29	-.14	-.13	.12	.05	-.05	.02 NS	.05	-.03 NS	-.08	-.07	—																					
**(22) Market rented housing**	-.03 NS	.07	.00 NS	-.01 NS	-.09	-.13	.21	-.11	-.14	.03 NS	-.04 NS	.01 NS	-.05	.06	-.04 NS	.01 NS	-.18	.00 NS	.12	-.20	-.16	—																				
**(23) Owned housing**	.38	.11	.15	.39	.45	.12	-.24	-.27	-.32	.41	.41	.29	-.28	-.09	.09	.04 NS	-.16	.42	-.13	-.21	-.17	-.49	—																			
**(24) Shared/other types of housing**	-.18	-.10	-.11	-.15	-.18	-.05	.08	.21	.40	-.21	-.25	-.14	.11	.04 NS	.01 NS	-.01 NS	.33	-.37	.13	-.15	-.12	-.36	-.37	—																		
**(25) Cohort member was born underweight**	-.09	-.02 NS	-.01 NS	-.08	-.10	-.04 NS	.06	.13	.15	-.14	-.05	-.07	.16	-.04 NS	-.06	-.10	.00 NS	-.07	.11	.09	.01 NS	.03 NS	-.17	.10	—																	
**(26) Cohort member's general health at 1yr**	.12	.05	.10	.11	.11	.01 NS	-.02 NS	.01 NS	-.01 NS	.15	.17	.11	.05 NS	-.21	.08	-.24	-.08	-.05	.09	.02 NS	-.02 NS	.05	.02 NS	-.08	-.08	—																
**(27) Mother's general health at 1yr**	.26	.16	.24	.15	.13	-.01 NS	-.01 NS	-.07	-.07	.25	.25	.13	.03 NS	-.15	-.02 NS	-.22	-.18	.03 NS	.08	.00 NS	-.09	-.01 NS	.19	-.16	.04 NS	.27	—															

Variables	(1)	(2)	(3)	(4)	(5)	(6)	(7)	(8)	(9)	(10)	(11)	(12)	(13)	(14)	(15)	(16)	(17)	(18)	(19)	(20)	(21)	(22)	(23)	(24)	(25)	(26)	(27)	(28)	(29)	(30)	(31)	(32)	(33)	(34)	(35)	(36)	(37)	(38)	(39)	(40)	(41)	(42)
**(28) Mother depressed at 1yr**	-.07	-.11	-.11	-.03 NS	-.06	-.04 NS	.11	.03 NS	.03 NS	-.05	-.09	.02 NS	.02 NS	-.06	.05	-.09	.03 NS	-.07	.00 NS	-.01 NS	.10	.05	-.13	.05	-.03 NS	-.07	-.23	—														
**(29) Household was workless at 5yrs**	-.22	-.06	-.06	-.06	-.12	-.10	.12	-.13	-.02 NS	-.10	-.19	-.06	.00 NS	-.01 NS	.14	-.04 NS	.04 NS	-.14	.02 NS	.04 NS	.02 NS	.11	-.21	.08	-.03 NS	.15	-.11	.21	—													
**(30) Household was employed at 5yrs**	-.15	-.13	-.08	-.28	-.21	.10	.06	.78	.36	-.32	-.21	-.14	.20	.00 NS	-.08	-.06	.07	-.24	.05	.16	.17	-.10	-.21	.16	.10	.01 NS	-.09	.03 NS	-.10	—												
**(31) Mother was single at 5yrs**	-.07	-.04 NS	-.07	-.04 NS	-.10	-.09	.14	-.09	-.23	-.09	-.15	-.08	.09	-.03 NS	.04 NS	-.05	.06	.03 NS	-.06	.02 NS	.02 NS	.11	-.12	-.02 NS	-.03 NS	.05	-.11	.25	.48	-.11	—											
**(32) Mother was coupled at 5yrs**	-.09	-.08	-.03 NS	-.19	-.19	.00 NS	.14	.28	.57	-.19	-.20	-.10	.18	-.04 NS	-.05	-.10	.14	-.26	.09	.08	.02 NS	-.12	-.19	.29	.08	-.02 NS	-.01 NS	.05	-.07	.35	-.13	—										
**(33) Change in LN equivalized income 1-5yrs**	-.02 NS	-.02 NS	.05	-.09	-.07	.02 NS	.04 NS	.14	.16	-.52	-.10	-.06	.05	.00 NS	.01 NS	.01 NS	.05	-.08	-.03 NS	.04 NS	.13	-.05	-.12	.10	.11	-.04 NS	-.07	.05	-.10	.20	-.12	.09	—									
**(34) Change in household size 1-5yrs**	.16	.03 NS	.04 NS	.15	.17	.04 NS	-.07	-.07	-.18	.19	.26	.15	-.07	-.10	.01 NS	-.08	-.59	.13	.09	.02 NS	.03 NS	.16	.12	-.36	-.06	.03 NS	.10	-.03 NS	-.07	-.08	-.14	-.14	-.07	—								
**(35) Cohort member's sex in male**	.01 NS	-.05	.01 NS	-.02 NS	-.01 NS	.00 NS	-.03 NS	-.02 NS	.00 NS	.06	.12	-.02 NS	.03 NS	-.02 NS	.01 NS	.00 NS	-.04 NS	.01 NS	.01 NS	.03 NS	-.06	-.02 NS	.04 NS	-.01 NS	.01 NS	-.12	.10	-.14	-.10	.00 NS	-.19	.01 NS	.02 NS	.00 NS	—							
**(36) Cohort member's age in months at 5yrs**	-.06	-.05	-.13	-.08	-.12	-.07	.11	.05	.04 NS	-.09	-.02 NS	-.12	.16	.01 NS	-.08	-.12	-.05	-.14	-.01 NS	.01 NS	-.01 NS	-.04 NS	-.01 NS	.06	-.05	.02 NS	.02 NS	.05	.01 NS	.04 NS	.11	.00 NS	.00 NS	.00 NS	.10	—						
(37) Area social advantage at 1yr (original score)	.41	.15	.14	.97	.75	-.30	-.01 NS	-.37	-.28	.53	.48	.50	-.43	-.22	.17	-.10	-.24	.29	.13	-.38	-.16	.04 NS	.37	-.14	-.09	.10	.13	-.04 NS	-.04 NS	-.32	-.03 NS	-.19	-.09	.14	-.02 NS	-.08	—					
(38) Change area social advantage 1-5yrs (difference of original scores)	.10	.00 NS	.06	-.25	.30	.85	-.45	.08	.00 NS	.00 NS	.09	.02 NS	.04 NS	-.03 NS	-.05	.08	.02 NS	.18	-.13	.05	.09	-.21	.21	-.08	-.06	.00 NS	-.03 NS	-.08	-.14	.06	-.12	-.10	.03 NS	.07	.03 NS	-.09	-.27	—				
(39) LN area social advantage (natural log)	.35	.13	.12	.87	.68	-.27	.00 NS	-.36	-.25	.47	.41	.44	-.42	-.16	.15	-.06	-.20	.25	.11	-.41	-.14	.07	.33	-.12	-.09	.09	.10	-.03 NS	-.03 NS	-.31	-.02 NS	-.18	-.09	.12	-.02 NS	-.07	.95	-.28	—			
(40) Change LN area social advan. 1-5yrs (difference of LNs)	.14	.04 NS	.07	-.14	.34	.74	-.29	.06	-.04 NS	.04 NS	.11	.07	-.02 NS	-.04 NS	-.03 NS	.06	-.04 NS	.18	-.10	.01 NS	.06	-.14	.17	-.08	-.04 NS	-.02 NS	-.04 NS	-.05	-.10	.04 NS	-.07	-.13	.05	.09	.02 NS	-.07	-.19	.90	-.28	—		
(41) Negative change area social advantage 1-5yrs (diff. p-tiles, p.=0)	.12	.03 NS	.07	-.25	.25	.76	-.34	.07	.03 NS	.00 NS	.09	.04 NS	.04 NS	-.02 NS	-.11	.07	.05	.18	-.13	.04 NS	.06	-.17	.19	-.08	-.02 NS	-.02 NS	.02 NS	-.12	-.17	.05	-.13	-.03 NS	-.01 NS	.02 NS	.04 NS	-.13	-.24	.79	-.22	.66	—	
(42) Positive change area social advantage 1-5yrs (diff. p-tiles, n.=0)	.06	-.04 NS	.01 NS	-.23	.30	.78	.33	.08	.09	-.01 NS	.02 NS	.01 NS	.00 NS	-.02 NS	.01 NS	.01 NS	-.05	-.03 NS	.04 NS	.01 NS	.07	-.03 NS	-.01 NS	.00 NS	-.04 NS	.03 NS	-.04 NS	.05	.01 NS	.10	-.01 NS	.03 NS	.04 NS	.05 NS	-.03 NS	.02 NS	-.22	.51	-.19	.49	.19	—

[a] Correlations are statistically significant at least at p < .05 unless noted as "Not (statistically) Significant" (NS).
[b] Additional variables used in the sensitivity analyses are listed in *italics* at the bottom of the table (see row 37ff).

Supplement S2: Full Tables for Multivariate Analyses

Table S2.1 Verbal Score at 5yrs: OLS Unstandardized Regressions Coefficients (Standard Error in parentheses) in UK (N=7,967) and US (N=1,458)

Variable	Model 1: Area Social Advantage		Model 2: Area and Mobility		Model 3: Model 2 + Family Context & Health		Model 4: Model 3 + Changes Family Context	
	UK	*US*	*UK*	*US*	*UK*	*US*	*UK*	*US*
Area social advantage at 1yr (percentile)	0.31^{***} (0.02)	0.47^{***} (0.09)	0.33^{***} (0.02)	0.58^{***} (0.06)	0.11^{***} (0.02)	0.10 (0.11)	0.10^{***} (0.02)	0.07 (0.10)
Change in area social advantage 1-5yrs (diff. of p-tiles)	—	—	0.16^{***} (0.02)	0.47^{**} (0.11)	0.04 (0.02)	0.23^{**} (0.08)	0.04 (0.02)	0.21^{**} (0.07)
Moved between 1-5yrs	—	—	-0.40 (0.87)	2.55 (5.53)	-0.91 (0.82)	1.99 (3.29)	-0.87 (0.83)	1.46 (3.29)
Household was workless at 1yr	—	—	—	—	-1.98 (1.16)	1.15 (4.49)	-3.10^{*} (1.16)	0.64 (6.25)
Mother was single at 1yr	—	—	—	—	1.04 (1.22)	-2.77 (4.47)	-0.01 (1.49)	-4.64 (4.74)
LN equivalized income at 1yr	—	—	—	—	3.18^{***} (0.86)	1.44^{**} (0.66)	3.82^{***} (0.94)	3.71^{**} (0.95)
Mother's level of education	—	—	—	—	2.67^{***} (0.24)	1.63 (1.02)	2.57^{***} (0.24)	0.98 (1.02)
Race/Ethnicity (ref. group: *White*)								
Black	—	—	—	—	-15.01^{***} (1.94)	-17.14^{***} (4.10)	-14.55^{***} (1.96)	-17.51^{**} (4.19)
Hispanic	—	—	—	—	—	-20.63^{***} (3.88)	—	-19.91^{***} (3.88)
Indian	—	—	—	—	-6.54^{**} (2.2)	—	-6.46^{**} (2.2)	—
Pakistani/Bangladeshi	—	—	—	—	-17.91^{***} (1.50)	—	-17.46^{***} (1.96)	—

Variable	Model 1: Area Social Advantage		Model 2: Area and Mobility		Model 3: Model 2 + Family Context & Health		Model 4: Model 3 + Changes Family Context	
	UK	*US*	*UK*	*US*	*UK*	*US*	*UK*	*US*
Other race/ethnicity	—	—	—	—	-14.07*** (1.74)	-3.18 (7.72)	-14.03*** (1.74)	-1.34 (6.98)
Mother was not born in UK/US	—	—	—	—	-4.15** (1.26)	-9.25*** (2.73)	-4.5** (1.27)	-9.18** (2.97)
Household size at 1yr	—	—	—	—	-1.92*** (0.35)	-1.66 (1.04)	-2.34*** (0.38)	-2.37* (1.02)
Mother's age in years at cohort member's birth	—	—	—	—	0.32*** (0.07)	0.43 (0.28)	0.31*** (0.07)	0.41 (0.28)
Cohort member was first child	—	—	—	—	4.01*** (0.86)	0.70 (2.86)	3.88*** (0.87)	-0.29 (2.66)
Housing Tenure at 1yr (ref. group: *Owned housing*)								
Public housing	—	—	—	—	-1.68 (1.00)	-10.49** (3.64)	-1.09 (1.07)	-8.22* (3.83)
Subsidized rented housing	—	—	—	—	-4.75* (2.07)	-10.85** (5.20)	-4.05 (2.13)	-8.00 (4.16)
Market rented housing	—	—	—	—	0.38 (1.68)	-3.60 (3.42)	0.58 (1.69)	-2.30 (3.32)
Shared/other types of housing	—	—	—	—	2.56 (1.86)	-4.60 (3.95)	1.84 (1.89)	-3.65 (3.80)
Cohort Member was born underweight	—	—	—	—	-1.43 (1.30)	-5.62 (3.72)	-1.38 (1.29)	-5.72 (3.66)
Cohort Member's general health at 3yrs/1yr	—	—	—	—	-2.93** (0.93)	-2.07 (1.68)	-2.98** (0.92)	-1.25 (1.85)
Mother's general health at 1yr	—	—	—	—	0.13 (0.60)	3.14** (0.92)	0.09 (0.60)	2.69* (0.93)
Mother depressed at 1yr	—	—	—	—	0.11 (0.87)	-0.86 (5.12)	0.14 (0.86)	-2.33 (5.05)

Variable	Model 1: Area Social Advantage		Model 2: Area and Mobility		Model 3: Model 2 + Family Context & Health		Model 4: Model 3 + Changes Family Context	
	UK	*US*	*UK*	*US*	*UK*	*US*	*UK*	*US*
Household was workless at 5yrs	—	—	—	—	—	—	-0.11 (1.46)	-12.54*** (2.55)
Household was employed at 5yrs	—	—	—	—	—	—	2.96 (1.56)	-0.89 (4.25)
Mother was single at 5yrs	—	—	—	—	—	—	-2.53* (1.05)	3.90 (2.11)
Mother was coupled at 5yrs	—	—	—	—	—	—	1.16 (1.72)	6.19 (4.15)
Change in LN equivalized income 1-5yrs	—	—	—	—	—	—	0.95 (0.79)	3.27* (1.17)
Change in household size 1-5yrs	—	—	—	—	—	—	-1.13** (0.39)	-1.16 (0.84)
Cohort Member's sex is male	-2.35** (0.71)	1.47 (2.44)	-2.29** (0.70)	1.51 (2.28)	-2.28** (0.66)	-1.83 (1.19)	-2.26** (0.66)	-2.35 (1.18)
Cohort Member's age in months at 5yrs	-0.34*** (0.13)	-0.28 (0.94)	-0.36** (0.12)	-0.23 (0.94)	-0.30* (0.10)	0.14 (0.58)	-0.29* (0.10)	0.13 (0.60)
**Constant**	59.41*** (7.89)	48.15 (60.05)	59.49*** (7.89)	10.22 (55.92)	25.81* (10.75)	31.15 (41.07)	22.08 (11.33)	15.66 (42.95)
**F-test**	104***	70***	70***	57***	133***	10,189***	119***	3,958***
**Adjusted R^2**	.10	.20	.11	.28	.24	.46	.24	.48

*p<.05; **p<.01; ***p<.001.

Table S2.2 Externalizing Behavior Adjustment at 5yrs: OLS Unstandardized Regressions Coefficients (Standard Error in parentheses) in UK (N=7,668) and US (N=1,820)

Variable	Model 1: Area Social Advantage		Model 2: Area and Mobility		Model 3: Model 2 + Family Context & Health		Model 4: Model 3 + Changes Family Context	
	UK	***US***	***UK***	***US***	***UK***	***US***	***UK***	***US***
Area social advantage at 1yr (percentile)	0.22*** (0.01)	0.15** (0.05)	0.23*** (0.01)	0.17** (0.05)	0.04* (0.02)	0.10 (0.10)	0.04* (0.02)	0.09 (0.10)
Change in area social advantage 1-5yrs (diff. of p-tiles)	—	—	0.07** (0.03)	0.07 (0.06)	-0.03 (0.03)	0.02 (0.05)	-0.04 (0.03)	0.03 (0.06)
Moved between 1-5yrs	—	—	2.46** (0.77)	3.71 (2.61)	-0.72 (0.78)	5.01** (1.51)	-0.62 (0.79)	5.09* (1.82)
Household was workless at 1yr	—	—	—	—	-2.03 (1.48)	1.07 (4.43)	-3.16 (1.82)	12.02 (6.34)
Mother was single at 1yr	—	—	—	—	-1.09 (1.62)	-1.71 (2.42)	-1.04 (1.93)	-4.72 (3.86)
LN equivalized income at 1yr	—	—	—	—	0.37 (0.99)	0.01 (1.08)	0.75 (1.33)	0.88 (1.46)
Mother's level of education	—	—	—	—	2.64*** (0.31)	-.04 (1.05)	2.56*** (0.31)	-.17 (1.23)
Race/Ethnicity (ref. group: *White*)								
Black	—	—	—	—	1.25 (1.99)	0.72 (3.71)	1.60 (1.93)	1.78 (3.66)
Hispanic	—	—	—	—	—	4.61 (9.02)	—	4.69 (8.81)
Indian	—	—	—	—	-1.52 (2.10)	—	-1.86 (2.12)	—
Pakistani/Bangladeshi	—	—	—	—	-3.79 (2.14)	—	-3.98 (2.12)	—
Other race/ethnicity	—	—	—	—	-0.39 (2.13)	-9.99 (6.45)	-0.24 (2.14)	-10.38 (6.17)

Variable	Model 1: Area Social Advantage		Model 2: Area and Mobility		Model 3: Model 2 + Family Context & Health		Model 4: Model 3 + Changes Family Context	
	UK	*US*	*UK*	*US*	*UK*	*US*	*UK*	*US*
Mother was not born in UK/US	—	—	—	—	2.32 (1.41)	-0.69 (2.30)	2.08 (1.41)	-1.03 (1.50)
Household size at 1yr	—	—	—	—	0.72 (0.36)	-0.62 (1.02)	0.91* (0.43)	-1.18 (1.29)
Mother's age in years at cohort member's birth	—	—	—	—	0.36*** (0.09)	0.63 (0.36)	0.36*** (0.09)	0.66 (0.38)
Cohort member was first child	—	—	—	—	0.40 (0.96)	2.37 (3.01)	0.54 (0.96)	2.35 (2.89)
Housing Tenure at 1yr (ref. group: *Owned housing*)								
Public housing	—	—	—	—	-5.09*** (1.38)	-3.54 (8.32)	-4.34** (1.42)	-3.26 (7.72)
Subsidized rented housing	—	—	—	—	-3.74 (2.37)	-7.88 (5.41)	-2.92 (2.44)	-6.47 (4.93)
Market rented housing	—	—	—	—	-2.93 (1.72)	0.95 (2.68)	-2.52 (1.75)	1.28 (2.58)
Shared/other types of housing	—	—	—	—	-1.69 (2.11)	-1.18 (4.22)	-1.21 (2.11)	-1.71 (4.55)
Cohort Member was born underweight	—	—	—	—	-3.65** (1.17)	-0.95 (5.90)	-3.60** (1.16)	-1.47 (6.01)
Cohort Member's general health at 3yrs/1yr	—	—	—	—	-2.61* (1.03)	0.60 (1.67)	-2.60* (1.04)	0.52 (1.70)
Mother's general health at 1yr	—	—	—	—	3.98*** (0.56)	3.20** (0.96)	3.85*** (0.58)	2.89* (0.94)
Mother depressed at 1yr	—	—	—	—	-2.75** (0.88)	-6.36 (6.55)	-2.53** (0.88)	-6.36 (6.92)
Household was workless at 5yrs	—	—	—	—	—	—	-1.88 (1.77)	2.80 (4.25)

Variable	Model 1: Area Social Advantage		Model 2: Area and Mobility		Model 3: Model 2 + Family Context & Health		Model 4: Model 3 + Changes Family Context	
	UK	***US***	***UK***	***US***	***UK***	***US***	***UK***	***US***
Household was employed at 5yrs	—	—	—	—	—	—	1.53 (2.06)	-15.81* (5.84)
Mother was single at 5yrs	—	—	—	—	—	—	-4.13** (1.45)	-4.10 (2.85)
Mother was coupled at 5yrs	—	—	—	—	—	—	-2.01 (1.91)	1.72 (5.67)
Change in LN equivalized income 1-5yrs	—	—	—	—	—	—	0.81 (0.98)	1.62 (1.20)
Change in household size 1-5yrs	—	—	—	—	—	—	0.32 (0.47)	-1.32 (0.97)
Cohort Member's sex is male	-7.63** (0.63)	-2.66 (1.92)	-7.62** (0.62)	-2.51 (2.01)	-7.63*** (0.60)	-3.88* (1.59)	-7.63*** (0.61)	-4.27* (1.70)
Cohort Member's age in months at 5yrs	0.26 (0.13)	-0.44 (0.39)	0.27 (0.13)	-0.47 (0.37)	0.26* (0.12)	-0.45 (0.41)	0.27* (0.12)	-0.37 (0.47)
**Constant**	32.72*** (8.48)	74.18** (23.21)	32.48*** (8.44)	72.97*** (22.40)	9.43 (11.26)	46.45 (23.53)	5.56 (13.53)	37.90 (26.46)
**F-test**	168***	6	103***	4*	52***	810***	44***	1,093***
**Adjusted R^2**	.06	.03	.07	.03	.13	.09	.13	.10

*p<.05; **p<.01; ***p<.001.

Table S2.3 Internalizing Behavior Adjustment at 5yrs: OLS Unstandardized Regressions Coefficients (Standard Error in parentheses) in UK (N=7,668) and US (N=1,820)

Variable	Model 1: Area Social Advantage		Model 2: Area and Mobility		Model 3: Model 2 + Family Context & Health		Model 4: Model 3 + Changes Family Context	
	UK	***US***	***UK***	***US***	***UK***	***US***	***UK***	***US***
Area social advantage at 1yr (percentile)	0.22^{***} (0.01)	0.16^{**} (0.05)	0.24^{***} (0.02)	0.19^{*} (0.07)	0.07^{**} (0.02)	0.15 (0.12)	0.06^{**} (0.02)	0.12 (0.13)
Change in area social advantage 1-5yrs (diff. of p-tiles)	—	—	0.12^{***} (0.03)	0.15 (0.11)	0.03 (0.03)	0.13 (0.11)	0.02 (0.03)	0.12 (0.12)
Moved between 1-5yrs	—	—	-1.54^{*} (0.75)	-0.32 (3.40)	-0.51 (0.73)	0.59 (3.59)	-0.53 (0.73)	0.32 (3.56)
Household was workless at 1yr	—	—	—	—	-2.76 (1.68)	0.59 (4.21)	-2.97 (1.91)	9.36 (5.65)
Mother was single at 1yr	—	—	—	—	-0.36 (1.50)	1.63 (1.57)	-0.74 (1.88)	-0.21 (2.54)
LN equivalized income at 1yr	—	—	—	—	2.77^{**} (0.87)	0.40 (1.32)	5.03^{***} (1.18)	3.62^{*} (1.60)
Mother's level of education	—	—	—	—	1.99^{***} (0.28)	0.86 (1.07)	1.80^{***} (0.29)	0.38 (0.99)
Race/Ethnicity (ref. group: *White*)								
Black	—	—	—	—	-4.27^{*} (1.85)	9.39^{*} (3.22)	-3.68^{*} (1.86)	10.30^{**} (3.22)
Hispanic	—	—	—	—	—	5.61 (8.42)	—	6.50 (8.54)
Indian	—	—	—	—	-4.55 (2.68)	—	-4.58 (2.69)	—
Pakistani/Bangladeshi	—	—	—	—	-12.00^{***} (2.05)	—	-11.44^{***} (2.08)	—
Other race/ethnicity	—	—	—	—	-8.35^{***} (2.22)	5.20 (7.95)	-8.11^{***} (2.22)	3.64 (7.54)

Variable	Model 1: Area Social Advantage		Model 2: Area and Mobility		Model 3: Model 2 + Family Context & Health		Model 4: Model 3 + Changes Family Context	
	UK	***US***	***UK***	***US***	***UK***	***US***	***UK***	***US***
Mother was not born in UK/US	—	—	—	—	0.14 (1.28)	-6.36 (5.77)	0.06 (1.26)	-5.90 (5.74)
Household size at 1yr	—	—	—	—	-0.86 (0.44)	0.42 (0.92)	-0.74 (0.52)	0.44 (0.97)
Mother's age in years at cohort member's birth	—	—	—	—	0.03 (0.09)	-0.29 (0.21)	0.01 (0.08)	-0.30 (0.24)
Cohort member was first child	—	—	—	—	-6.60*** (1.01)	-1.31 (1.72)	-6.53*** (1.02)	-1.42 (2.06)
Housing Tenure at 1yr (ref. group: *Owned housing*)								
Public housing	—	—	—	—	-1.97 (1.38)	-10.52 (5.61)	-1.18 (1.46)	-9.99 (5.70)
Subsidized rented housing	—	—	—	—	3.83 (2.89)	-10.09 (6.51)	4.83 (2.96)	-8.45 (6.88)
Market rented housing	—	—	—	—	1.16 (2.06)	-3.08 (5.44)	1.62 (2.10)	-2.82 (6.06)
Shared/other types of housing	—	—	—	—	4.83* (2.26)	-9.44 (5.18)	4.91* (2.30)	-10.26 (5.49)
Cohort Member was born underweight	—	—	—	—	-2.13 (1.46)	-1.05 (4.80)	-2.06 (1.43)	-1.62 (5.14)
Cohort Member's general health at 3yrs/1yr	—	—	—	—	-2.72* (1.23)	0.15 (1.16)	-2.71* (1.25)	-0.09 (1.23)
Mother's general health at 1yr	—	—	—	—	4.50*** (0.56)	5.11*** (1.14)	4.37*** (0.57)	4.79** (1.26)
Mother depressed at 1yr	—	—	—	—	-3.70*** (0.98)	-4.08 (6.20)	-3.54*** (0.98)	-5.45 (5.70)
Household was workless at 5yrs	—	—	—	—	—	—	0.50 (9.88)	2.89 (3.98)

Variable	Model 1: Area Social Advantage		Model 2: Area and Mobility		Model 3: Model 2 + Family Context & Health		Model 4: Model 3 + Changes Family Context	
	UK	***US***	***UK***	***US***	***UK***	***US***	***UK***	***US***
Household was employed at 5yrs	—	—	—	—	—	—	1.37 (1.89)	-11.19* (5.09)
Mother was single at 5yrs	—	—	—	—	—	—	-1.62 (1.56)	-0.05 (3.62)
Mother was coupled at 5yrs	—	—	—	—	—	—	0.68 (2.27)	3.06 (4.54)
Change in LN equivalized income 1-5yrs	—	—	—	—	—	—	3.41*** (0.95)	4.81*** (1.10)
Change in household size 1-5yrs	—	—	—	—	—	—	-0.06 (0.49)	-0.44 (1.08)
Cohort Member's sex is male	-0.66 (0.72)	1.24 (2.69)	-0.63 (0.72)	1.18 (2.73)	-0.76 (0.72)	-0.84 (2.54)	-0.72 (0.72)	-1.22 (2.16)
Cohort Member's age in months at 5yrs	0.12 (0.15)	-1.77* (0.73)	0.11 (0.15)	-1.63* (0.75)	0.10 (0.15)	-2.09** (0.56)	0.11 (0.15)	-1.96** (0.53)
**Constant**	42.04*** (9.56)	152.90** (45.57)	41.96*** (9.59)	142.93** (45.57)	14.88 (13.75)	154.37*** (32.21)	-6.22 (16.79)	119.65** (29.66)
**F-test**	74***	12***	45***	12***	42***	2,571***	38***	3,447***
**Adjusted R^2**	.04	.04	.05	.05	.10	.11	.10	.13

*p<.05; **p<.01; ***p<.001.

Supplement S3: Plots of Predicted Margins on "Area and Mobility" Variables

Figure S3.1 Plots of Linear Prediction of Margins (95% CIs) for Dependent Variables in UK and US by Model and Variable of the "Area and Mobility" Domain

		Model 2: Area and Mobility			Model 3: Model 2 + Family Context & Health			Model 4: Model 3 + Changes in Family Context		
		Area social advantage at 1yr	*Change in area social advantage 1-5yrs*	*Moved between 1-5yrs*	*Area social advantage at 1yr*	*Change in area social advantage 1-5yrs*	*Moved between 1-5yrs*	*Area social advantage at 1yr*	*Change in area social advantage 1-5yrs*	*Moved between 1-5yrs*
**Verbal Score at 5yrs**	***UK***									
	US									
**Externalizing Behavior Adjustment at 5yrs**	***UK***									
	US									
**Internalizing Behavior Adjustment at 5yrs**	***UK***									
	US									

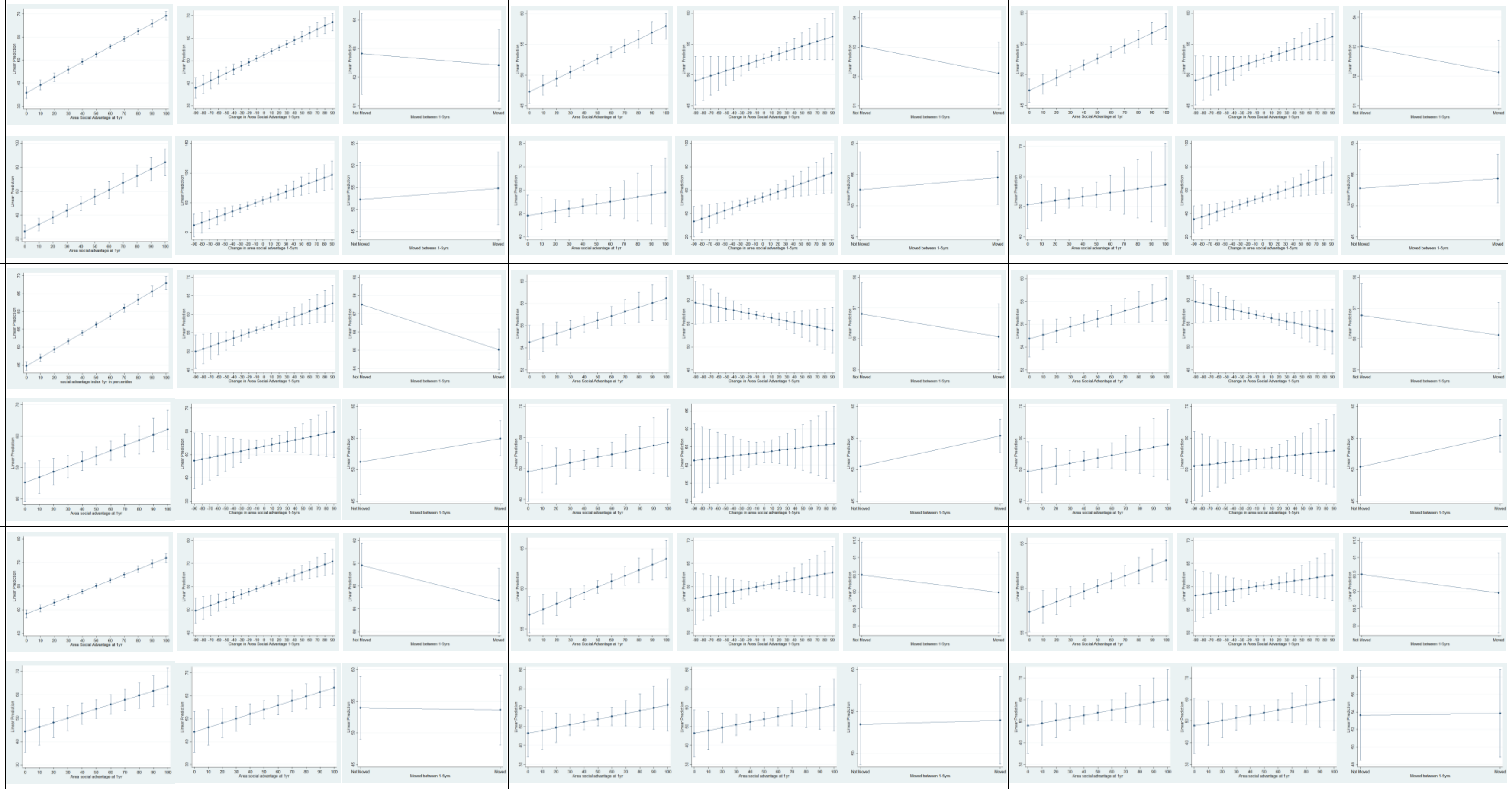

Supplement S4: Sensitivity Analyses

Table S4.1 - Comparison of OLS Unstandardized Regressions Coefficients (Standard Errors in parentheses) on Dependent Variables in UK for Different Versions of "Area Social Advantage and (Relative) Change" - Selection of Parameters from "Model 4: Changes in Family Context"[a]

Variables	Verbal Score at 5yrs (N=7,967)				Externalizing behavior adjustment at 5yrs (N=7,668)				Internalizing behavior adjustment at 5yrs (N=7,668)			
	Percentile	*Change: Split P-tile*	*LN*	*Original*	*Percentile*	*Change: Split P-tile*	*LN*	*Original*	*Percentile*	*Change: Split P-tile*	*LN*	*Original*
Area social advantage at 1yr (percentile)	0.10*** (0.02)	0.10*** (0.02)	—	—	0.04* (0.02)	0.04* (0.02)	—	—	0.06** (0.02)	0.06** (0.02)	—	—
Change in area social advantage 1-5yrs (difference of percentiles)	0.04 (0.02)	—	—	—	-0.04 (0.03)	—	—	—	0.02 (0.03)	—	—	—
Negative change in area social advantage 1-5yrs (difference of percentile, positive values = 0)	—	-0.01 (0.04)	—	—	—	-0.03 (0.05)	—	—	—	0.01 (0.05)	—	—
Positive change in area social advantage 1-5yrs (difference of percentiles, negative values = 0)	—	0.08* (0.03)	—	—	—	-0.04 (0.04)	—	—	—	0.04 (0.04)	—	—
LN area social advantage at 1yr (natural log)	—	—	6.92*** (1.38)	—	—	—	2.68* (1.15)	—	—	—	3.67* (1.43)	—
Change in LN area social advantage 1-5yrs (difference of LNs)	—	—	2.02 (1.59)	—	—	—	-0.74 (1.99)	—	—	—	2.54 (2.14)	—
Area social advantage at 1yr (original score)	—	—	—	2.64*** (0.48)	—	—	—	0.92* (0.42)	—	—	—	1.52** (0.52)
Change in area social advantage 1-5yrs (difference of original scores)	—	—	—	0.97 (0.56)	—	—	—	-0.82 (0.70)	—	—	—	0.79 (0.78)
Moved between 1–5yrs	-0.87 (0.83)	-1.69 (1.07)	-0.83 (0.84)	-0.93 (0.83)	-0.62 (0.79)	-0.51 (1.02)	-0.67 (0.79)	-0.60 (0.79)	-0.53 (0.73)	-0.74 (0.92)	-0.57 (0.73)	-0.60 (0.74)
**Constant**	22.08 (11.33)	21.53 (11.40)	10.18 (11.20)	25.62 (11.42)	5.56 (13.53)	5.64 (13.54)	1.76 (13.06)	7.12 (13.71)	-6.22 (16.79)	-6.35 (16.70)	-13.06 (16.27)	-3.92 (16.93)
**F-test**	119***	114***	109***	115***	44***	43***	46***	45***	38***	37***	38***	39***
**Adjusted R^2**	.24	.24	.24	.24	.13	.13	.13	.13	.10	.10	.10	.10

*p<.05; **p<.01; ***p<.001.

[a] Shaded columns refer to the specification used in the study.

Table S4.2 - Comparison of OLS Unstandardized Regressions Coefficients (Standard Errors in parentheses) on Dependent Variables in US for Different Versions of "Area Social Advantage and (Relative) Change" - Selection of Parameters from "Model 4: Changes in Family Context"[a]

Variables	Verbal Score at 5yrs (N=1,458)				Externalizing behavior adjustment at 5yrs (N=1,820)				Internalizing behavior adjustment at 5yrs (N=1,820)			
	Percentile	*Change: Split P-tile*	*LN*	*Original*	*Percentile*	*Change: Split P-tile*	*LN*	*Original*	*Percentile*	*Change: Split P-tile*	*LN*	*Original*
Area social advantage at 1yr (percentile)	0.07 (0.10)	0.08 (0.10)	—	—	0.09 (0.10)	0.09 (0.10)	—	—	0.12 (0.13)	0.13 (0.13)	—	—
Change in area social advantage 1-5yrs (difference of percentiles)	0.21** (0.07)	—	—	—	0.03 (0.06)	—	—	—	0.12 (0.12)	—	—	—
Negative change in area social advantage 1-5yrs (difference of percentile, positive values = 0)	—	0.40** (0.10)	—	—	—	0.19 (0.18)	—	—	—	0.19 (0.21)	—	—
Positive change in area social advantage 1-5yrs (difference of percentiles, negative values = 0)	—	0.05 (0.08)	—	—	—	-0.11 (0.13)	—	—	—	0.06 (0.10)	—	—
LN area social advantage at 1yr (natural log)	—	—	3.81 (7.62)	—	—	—	5.92 (5.94)	—	—	—	7.53 (7.67)	—
Change in LN area social advantage 1-5yrs (difference of LNs)	—	—	13.40** (4.15)	—	—	—	8.94 (5.19)	—	—	—	11.43 (8.46)	—
Area social advantage at 1yr (original score)	—	—	—	1.13 (2.12)	—	—	—	1.59 (1.84)	—	—	—	2.27 (2.41)
Change in area social advantage 1-5yrs (difference of original scores)	—	—	—	4.32* (1.52)	—	—	—	1.43 (1.36)	—	—	—	2.95 (2.49)
Moved between 1–5yrs	1.46 (3.29)	4.50 (3.38)	3.65 (3.30)	5.33 (3.68)	5.09* (1.82)	7.57 (3.91)	5.09* (1.82)	6.22* (2.67)	0.32 (3.56)	1.40 (3.30)	2.01 (4.16)	2.92 (4.82)
**Constant**	15.66 (42.95)	6.27 (41.21)	17.85 (45.46)	16.62 (41.65)	37.90 (26.46)	31.26 (27.40)	30.44 (23.09)	39.54 (27.95)	119.65** (29.66)	116.77** (31.42)	112.65** (29.64)	122.73** (29.91)
**F-test**	3,958***	3,595***	4,024***	3,869***	1,093***	1,413***	826***	930***	3,447***	4,427***	2,922***	3,060***
**Adjusted R^2**	.48	.49	.48	.48	.10	.11	.11	.10	.13	.13	.13	.13

*p<.05; **p<.01; ***p<.001.

[a] Shaded columns refer to the specification used in the study.
